# Development of Real-Time PCR Methods for the Detection of Bacterial Meningitis Pathogens without DNA Extraction

**DOI:** 10.1371/journal.pone.0147765

**Published:** 2016-02-01

**Authors:** Jeni Vuong, Jean-Marc Collard, Melissa J. Whaley, Issaka Bassira, Issaka Seidou, Seydou Diarra, Rasmata T. Ouédraogo, Dinanibè Kambiré, Thomas H. Taylor, Claudio Sacchi, Leonard W. Mayer, Xin Wang

**Affiliations:** 1 Meningitis and Vaccine Preventable Diseases Branch, Centers for Disease Control and Prevention, Atlanta, United States of America; 2 Scientific Institute of Public Health, Brussels, Belgium; 3 Centre de Recherche Médicale et Sanitaire, Niamey, Niger; 4 Institut National de Recherche en Santé Publique, Bamako, Mali; 5 Centre Hospitalier Universitaire Pédiatrique Charles De Gaulle, Ouagadougou, Burkina Faso; 6 Instituto Adolfo Lutz, São Paulo, Brazil; California Department of Public Health, UNITED STATES

## Abstract

*Neisseria meningitidis* (Nm), *Haemophilus influenzae* (Hi), and *Streptococcus pneumoniae* (Sp) are the lead causes of bacterial meningitis. Detection of these pathogens from clinical specimens using traditional real-time PCR (rt-PCR) requires DNA extraction to remove the PCR inhibitors prior to testing, which is time consuming and labor intensive. In this study, five species-specific (Nm-*sodC* and -*ctrA*, Hi-*hpd#*1 and -*hpd*#3 and Sp-*lytA*) and six serogroup-specific rt-PCR tests (A, B, C, W, X, Y) targeting Nm capsular genes were evaluated in the two direct rt-PCR methods using PerfeCTa and 5x Omni that do not require DNA extraction. The sensitivity and specify of the two direct rt-PCR methods were compared to TaqMan traditional rt-PCR, the current standard rt-PCR method for the detection of meningitis pathogens. The LLD for all 11 rt-PCR tests ranged from 6,227 to 272,229 CFU/ml for TaqMan, 1,824–135,982 for 5x Omni, and 168–6,836 CFU/ml for PerfeCTa. The diagnostic sensitivity using TaqMan ranged from 89.2%-99.6%, except for NmB-*csb*, which was 69.7%. For 5x Omni, the sensitivity varied from 67.1% to 99.8%, with three tests below 90%. The sensitivity of these tests using PerfeCTa varied from 89.4% to 99.8%. The specificity ranges of the 11 tests were 98.0–99.9%, 97.5–99.9%, and 92.9–99.9% for TaqMan, 5x Omni, and PerfeCTa, respectively. PerfeCTa direct rt-PCR demonstrated similar or better sensitivity compared to 5x Omni direct rt-PCR or TaqMan traditional rt-PCR. Since the direct rt-PCR method does not require DNA extraction, it reduces the time and cost for processing CSF specimens, increases testing throughput, decreases the risk of cross-contamination, and conserves precious CSF. The direct rt-PCR method will be beneficial to laboratories with high testing volume.

## Introduction

*Neisseria meningitidis* (Nm), *Haemophilus influenzae* (Hi), and *Streptococcus pneumoniae* (Sp) are common bacterial meningitis pathogens [1–3]. Traditional real-time PCR (rt-PCR) tests with high sensitivity and specificity have been implemented in many public health laboratories for the detection of these three pathogens [4–8]. Traditional rt-PCR uses DNA polymerase whose activity can be negatively affected by PCR inhibitors in clinical specimens [9–10]. DNA extraction is needed to remove the inhibitors to improve the sensitivity and specificity of traditional rt-PCR. Extracting DNA from clinical specimens is not only expensive but also labor-intensive. A well trained microbiologist can only process up to 40 specimens per day. Because multiple manipulation steps are involved, cross-contamination is often introduced during DNA extraction. In addition, the extraction process may not remove all of the inhibitors or recover 100% of the target DNA [10], which can reduce the diagnostic sensitivity and leads to false-negative results.

Direct rt-PCR uses modified DNA polymerases whose activity can overcome PCR inhibitors present in clinical matrices. These enzymes allow amplification of the target genes directly from clinical specimens without the need to extract DNA [11]. In this study, one traditional and two direct real-time PCR methods were evaluated for detecting *N*. *meningitidis* and its 6 disease-causing serogroups (A, B, C, W, X and Y), *H*. *influenzae*, and *S*. *pneumoniae*. We compared the sensitivity and specificity of the two new direct rt-PCR methods along with our current traditional rt-PCR method.

## Materials and Methods

### Ethics Statement

Ethics approval was not obtained from the CDC Institutional Review Board (IRB) for clinical specimens reported in this study because the specimens sent to CDC were existing specimens collected for clinical purposes. CDC investigators had no access to any identifiable private information about people. CDC IRB approval was not sought for cerebral spinal fluid specimens from Brazil, Burkina Faso, Mali and Niger specimens because these cerebral spinal fluid specimens were not considered human specimens, as they do not meet the definition of a living human subject. These specimens have been tested at the respective countries as part of their routine surveillance. These specimens were not collected specifically for this study.

### Study Design

The traditional rt-PCR method used the TaqMan^®^ Universal PCR master mix (Applied Biosystems, Foster City, CA) and extracted DNA as template. Traditional singleplex rt-PCR tests targeting 11 genes, have been published and implemented in many domestic and international public health laboratories (S1 Table) [4–8]. These 11 tests are considered as the standard rt-PCR method for detecting *N*. *meningitidis* and its 6 main disease-causing serogroups (A, B, C, W, X and Y), *H*.*influenzae*, and *S*. *pneumoniae*.

The two direct rt-PCR methods used either the 5x Omni Klentaq LA PCR Kit (DNA Polymerase Technology, St. Louis, MO) or PerfeCTa qPCR ToughMix (Quanta Biosciences, Gaithersburg, MD) to amplify the 11 target genes directly from CSF in singleplex reactions. The methods are described below.

Real-time PCR tests targeting each of the 11 genes for the pathogens described above were evaluated for both direct rt-PCR methods. The name of each PCR test include its target species or serogroup and the gene. Final concentration of primers and probe for each rt-PCR test was optimized by testing in the ranges of 100–900 nM for primers and 100–300 nM for probe (Table 1). Each rt-PCR reaction was prepared in a final volume of 25μl, containing 2 μl of CSF (S2 Table), and tested on the Stratagene Mx3005P (Agilent Technology, Santa Clara, CA) using the cycling conditions described in S3 Table.

**Table 1:** Final concentrations of primers and probes for the real-time PCR tests for detection of *Neisseria meningitidis* and its 6 serogroups, *Haemophilus influenzae*, and *Streptococcus pneumoniae* using traditional and direct methods^a^.

	Traditional	Direct	Direct
	TaqMan	5x Omni	PerfeCTa
PCR tests	(Fw/Rv/Pb nM)^a^	(Fw/Rv/Pb nM)	(Fw/Rv/Pb nM)
**Nm-*ctrA***	300/900/100	900/600/100	300/600/200
**Nm-*sodC***	300/600/100	Not compatible	300/300/200
**Hi-*hpd* #1**	300/100/200	900/300/900	600/600/300
**Hi-*hpd* #3**	100/300/100	Not compatible	100/900/300
**Sp-*lytA***	200/200/200	600/600/600	300/600/200
**NmA-*csaB***	300/900/100	900/600/100	900/600/300
**NmB-*csb***	300/300/100	600/900/200	900/300/200
**NmC-*csc***	900/300/100	900/300/300	300/300/300
**NmW-*csw***	100/900/200	600/600/200	300/100/200
**NmX-*csxB***	900/900/100	600/600/200	600/600/200
**NmY-*csy***	900/600/100	600/900/300	900/300/300

^a^ Fw = Forward primer, Rv = Reverse primer; Pb = Probe

To determine the lower limit of detection (LLD), target bacterial pathogens were first suspended in Brian Heart Infusion (BHI) broth, which was then serially diluted with CSF and BHI broth in 10-fold increments. The BHI dilutions (10^−5^–10^−7^) were plated onto their respective media and incubated at 35°C with 5% CO_2_ for 24 hours for enumeration of colony forming units per milliliter (CFU/ml). The CSF dilutions (10^−1^–10^−7^) were tested with direct rt-PCR using 5x Omni and PerfeCTa. To assess the LLD by traditional PCR method using ABI master mix, DNA was extracted from 200 μl of the 10^−1^ CSF dilution, eluted in 100 μl of elution buffer, and was then serially diluted with PCR grade water in 10-fold increments using QIAamp DNA Mini Kit (QIAGEN, Valencia, California). Traditional rt-PCR was performed on DNA dilutions (10^−1^–10^−7^). Each CSF or DNA dilution was tested by rt-PCR in triplicates. The LLD was defined as the number of CFU/ml that yielded a cycle threshold (C_t_) value of 35.

To determine the diagnostic sensitivity and specificity of the traditional and direct rt-PCR methods, CSF from meningitis patients were obtained from the following countries as part of their routine surveillance collection: Mali (n = 113), Burkina Faso (n = 131), Brazil (n = 82), Niger (n = 53), and the United States (n = 42). These CSFs had been tested previously at the respective countries using various microbiological tests such as culture, latex agglutination, counterimmunoelectrophoresis, and/or PCR. As these microbiological tests were not standardized in all countries and their sensitivity and specificity vary from country to country, none of the results from these tests were used as the reference standard due to their imperfect sensitivity and specificity. As a result, the diagnostic sensitivity and specificity were calculated using latent class analysis (LCA) which uses an internal reference standard based on the statistical model [12–13]. LCA uses data from at least 3 conditionally independent methods to determine the sensitivity and specificity without taking into consideration the laboratories’ results from the microbiological tests described above [12]. Since 5x Omni direct real-time PCR was not compatible for Nm-*sodC* andHi-*hpd* #3 tests, the laboratories’ results were used as the reference standard to obtain sensitivity and specificity using conventional 2 x 2 tables.

## Results and Discussion

The LLD for all 11 rt-PCR tests ranged from 6,227 to 272,229 CFU/ml for TaqMan traditional rt-PCR, 1,824–135,982 for 5x Omni direct rt-PCR, and 168–6,836 CFU/ml for PerfeCTa direct rt-PCR (Table 2). The diagnostic sensitivity of the 11 tests using TaqMan traditional rt-PCR ranged from 89.2%-99.6%, except for NmB-*csb*, which was 69.7% (Table 3). For 5x Omni direct rt-PCR, the sensitivity varied from 67.1% to 99.8%, with three tests below 90%. The sensitivity of these tests using PerfeCTa direct rt-PCR varied from 89.4% to 99.8%. The specificity ranges of the 11 tests were 98.0–99.9%, 97.5–99.9%, and 92.9–99.9% for TaqMan traditional rt-PCR, 5x Omni direct rt-PCR, and PerfeCTa direct rt-PCR, respectively (Table 3). Because Nm-*sodC* and Hi-*hpd* #3 tests were unable to amplify their target genes using 5x Omni direct rt-PCR, their sensitivity and specificity could not be calculated using LCA, which requires the results of at least 3 methods [13]. Therefore, the diagnostic sensitivity and specificity of Nm-*sodC* and Hi-*hpd* #3 were calculated using the non-PCR tests (culture, latex, or counterimmunoelectrophoresis) as the composite reference standard. The diagnostic sensitivity of the Nm-*sodC* assay was 100% for TaqMan traditional rt-PCR and PerfeCta direct rt-PCR methods; the diagnostic specificity was 96% and 92% for TaqMan traditional rt-PCR and PerfeCta direct rt-PCR methods, respectively. The diagnostic sensitivity of Hi-*hpd* #3 was 83% and 84.9% for TaqMan traditional rt-PCR and PerfeCta direct rt-PCR methods, respectively, and the diagnostic specificity was 100% for both methods.

**Table 2:** Lower limits of detection for the real-time PCR tests using traditional and direct methods.^a^

	Traditional	Direct	Direct
PCR Tests	TaqMan (CFU/ml)	5x Omni (CFU/ml)	PerfeCTa (CFU/ml)
**Nm-*ctrA***	24,098	5,145	197
**Nm-*sodC***	18,951	Not compatible	329
**Hi-*hpd* #1**	60,792	39,818	3,177
**Hi-*hpd* #3**	6,227	Not compatible	954
**Sp-*lytA***	63,861	86,755	6,836
**NmA-*csaB***	19,754	6,341	906
**NmB-*csb***	178,021	28,392	1,606
**NmC-*csc***	10,089	135,982	925
**NmW-*csw***	272,229	10,365	610
**NmX-*csxB***	9,618	27,699	174
**NmY-*csy***	73,264	1,824	168

^a^ Bacterial suspensions of known concentrations were prepared in Brain and Heart Infusion (BHI) broth. Ten-fold serial dilutions of BHI bacterial suspension were prepared with BHI broth and cerebrospinal fluid (CSF) for enumeration of colony forming units per milliliter (CFU/ml) and PCR testing in triplicate, respectively. Spiked CSF dilutions were tested by direct real-time PCR using 5x Omni and PerfeCTa master mix, whereas extracted DNA from the same spiked CSF dilutions was tested by traditional real-time PCR using TaqMan master mix. The CFU/ml of each BHI dilution was calculated from enumeration and plotted against the mean cycle threshold for each dilution based on the PCR triplicate testing. The lower limit of detection is the bacterial concentration yielding a C_t_ = 35.

**Table 3:** Diagnostic sensitivity and specificity for the real-time PCR tests by latent class analysis.

		Sensitivity (95% CI)				Specificity (95% CI)		
PCR		Traditional	Direct	Direct		Traditional	Direct	Direct
Tests	N^a^	TaqMan	5x Omni	PerfeCTa	N^a^	TaqMan	5x Omni	PerfeCTa
**Nm-*ctrA***	78	99.6% (95.5–100)	99.8% (95.5–100)	99.8% (95.5–100)	50	99.3% (92.5–100)	97.5% (88.7–99.9)	93.3% (82.5–98.7)
**Nm-*ctrA***^b^	78	99.8% (95.5–100)	99.8% (95.5–100)	99.8% (95.5–100)	48	99.3% (92.5–100)	99.3% (88.7–99.9)	99.3% (85.5–99.5)
**Nm-*sodC***^c^	77	NA	Not compatible	NA	50	NA	Not compatible	NA
**Hi-*hpd* #1**	53	96.2% (87.4–99.9)	96% (87.4–99)	99.2% (91.6–100)	50	98.1% (91.2–100)	99.7% (94.1–100)	95.3% (86.3–99)
**Hi-*hpd* #3**^c^	53	NA	Not compatible	NA	50	NA	Not compatible	NA
**Sp-*lytA***	114	98.8% (94.7–100)	92.9% (86.4–97.2)	99.7% (96.4–100)	50	98% (91.3–100)	99.5% (94.2–100)	97.9% (91.3–100)
**NmA-*csaB***	8	94.9% (63.1–100)	94.9% (63.1–100)	94.9% (63.1–100)	50	99.9% (92.9–100)	99.9% (92.9–100)	99.9% (92.9–100)
**NmB-*csb***	38	69.7% (53–84.1)	80.4% (64.8–92)	99.2% (90.5–100)	50	99.7% (93–100)	99.7% (93–100)	99.6% (93–100)
**NmC-*csc***	62	99.6% (94.1–100)	99.6% (94.1–100)	99.6% (94.1–100)	50	99.5% (93–100)	99.5% (93–100)	99.5% (93–100)
**NmW-*csw***	42	89.2% (75.8–97.1)	68.8% (51.3–82.5)	99.3% (91–100)	50	99.6% (93.2–100)	99.7% (93.2–100)	92.9% (84.1–98.8)
**NmW-*csw***^b^	42	89.2% (75.8–97.1)	68.8% (52.4–83)	99.3% (91–100)	47	99.6% (93.3–100)	99.7% (93.3–100)	99.5% (84.3–98.8)
**NmX-*csxB***	31	99% (88.8–100)	95.8% (83.3–99.9)	99% (88.4–100)	50	99.6% (92.9–100)	99.6% (92.9–100)	97.6% (89.4–99.9)
**NmY-*csy***	5	89.4% (39.8–100)	67.1% (19.4–99.4)	89.4% (39.8–100)	50	99.9% (93–100)	99.9% (93–100)	99.9% (93–100)

^a^ N = number of cerebrospinal fluid specimens tested. All specimens were collected from meningitis patients.

^b^ Recalculated diagnostic specificity by latent class analysis after exclusion of four specimens that were positive by direct real-time PCR using PerfeCTa but negative by traditional real-time PCR using TaqMan and direct real-time PCR using 5x Omni (Table 4).

^c^ NA = Since direct real-time PCR using 5x Omni was not compatible with these targets, Latent Class Analysis (LCA) could not be performed on these tests. Instead, the sensitivity and specificity of these tests were calculated using non-PCR tests as the reference standard as described in the text.

Although culture is considered the gold standard, culture is not always a perfect reference standard for calculating the diagnostic sensitivity and specificity due to the low culture rate (high false negative rate) in many regions. In contrast, LCA has become a preferred method for comparing the sensitivity and specificity for three or more independent methods. True positive and negative results are defined by the consensus of the methods that were evaluated instead of the reference standard, whose low sensitivity can directly affect the results of the evaluated methods. PerfeCTa direct rt-PCR detected more positives than TaqMan traditional and 5x Omni direct rt-PCR methods, which explains the lower specificity of the PerfeCTa NmW-*csw* and Nm-*ctrA* tests (92.9% and 93.3%, respectively). This was attributed to a few specimens that were negative by TaqMan and 5x Omni rt-PCR methods, yet were positive for one or more test(s) using PerfeCTa (Table 4). A specimen containing encapsulated Nm should be positive for *sodC* or *ctrA* and the serogroup specific gene. The presence of this pattern (Table 4) suggests that the PerfeCTa results were true positives while both the TaqMan and 5x Omni results were falsely negative. A revised LCA analysis excluding those specimens produced a specificity of 99.3% for Nm-*ctrA* and ~99.6% for NmW-*csw* for all 3 methods (Table 3).

**Table 4:** Cerebrospinal fluid specimens that were positive by direct real-time PCR using PerfeCTa and negative by traditional real-time PCR using TaqMan or direct real-time PCR using 5x Omni.

Specimen	Test	Lab ID	TaqMan	5x Omni	PerfeCTa
**1**	NmW-*csw*	M26789	Negative	Negative	Positive^a^
	Nm-*ctrA*	M26789	Negative	Positive	Positive^b^
**2**	NmW-*csw*	M26790	Negative	Negative	Positive^a^
	Nm-*ctrA*	M26790	Positive	Positive	Positive^b^

^a^ Two NmW-*csw* specimens tested positive only by PerfeCTa yet were negative by both TaqMan and 5x Omni.

^b^ Since the specimens were also positive by a second PCR target, Nm-*ctrA*, this provides additional assurance that the specimens are positive for NmW-*csw* using PerfeCTa.

While additional chemical, physical, or enzymatic lysis is recommended to increase DNA yield from gram-positive bacteria such as Sp and improve PCR diagnostic sensitivity and specificity, our data demonstrate that the PerfeCTa direct rt-PCR was able to detect Sp without compromising sensitivity. Although only a small number of positive CSF specimens for NmA-*csaB* (n = 8) and NmY-*csy* (n = 5) serogroups were tested, the results suggest that PerfeCTa will perform comparably for the detection of these two serogroups since PerfeCTa direct rt-PCR detects other serogroups with good sensitivity (Table 3). However, additional evaluation on a larger number of CSF specimens will be required to better assess the sensitivity and specify of the two tests.

PerfeCTa direct rt-PCR did not cross-detect other bacterial species (S4 Table) except for a veterinary pathogen, *Actinobacillus pleuropneumoniae*, which was positive for Nm-*ctrA* (C_t_ = 33) and Nm-*sodC* (C_t_ = 35), and *Aggregatibacter aphrophilus*, which was positive for *hpd*. These results were also previously observed with traditional rt-PCR [7]. *A*. *aphrophilus* has been implicated with causing brain abscesses [14]; association with meningitis has not been reported. While this is a limitation of the assay, this situation is rarely encountered as these pathogens are unlikely to be present in human CSF.

Real-time PCR has been implemented in clinical laboratories for the detection of bacterial meningitis pathogens. In regions with high burdens of disease, laboratories are required to test thousands of CSF specimens while trying to provide timely laboratory results. Extracting DNA from 20 CSF specimens takes about 3 or more hours, which is the bottleneck of traditional rt-PCR. Direct rt-PCR allows the detection of bacterial pathogens directly from CSF specimens without DNA extraction thereby reducing processing time, cost, labor, and risk of cross-contamination. Direct rt-PCR improves the testing throughput and provides a more robust method for the laboratories with high volume of specimens. Because there is no DNA loss with the elimination of DNA extraction, lower numbers of bacterial cells are detected compared to the traditional rt-PCR, well within the range detected by culture [15]. In addition, direct rt-PCR conserves precious CSF specimens as it only requires 2 μl of CSF per reaction.

In conclusion, we have demonstrated that the direct rt-PCR method detects meningitis pathogens with similar or better sensitivity and can replace the current traditional rt-PCR to improve the laboratory testing capacity. To further improve the PCR testing efficiency, we are currently validating direct rt-PCR in multiplex for the detection of bacterial meningitis pathogens and their serogroup and serotypes.

## Supporting Information

S1 Table Primers and probes used for rt-PCR assays. (DOCX)

S2 Table Reaction assembly for traditional and two direct real-time PCR methods. (DOCX)

S3 Table Cycling conditions for the traditional and two direct real-time PCR methods. (DOCX)

S4 Table Panel of 25 species representing 14 different genera of bacterial isolates used for cross-reactivity testing. (DOCX)
