# Supplementary material for: Development of Real-Time PCR Methods for the Detection of Bacterial Meningitis Pathogens without DNA Extraction
Source: PLoS One. 2016 Feb 1;11(2):e0147765. doi: 10.1371/journal.pone.0147765 (PMC4735509; doi:10.1371/journal.pone.0147765)
Supplement: S1 Table — (DOCX) [file pone.0147765.s001.docx]

**Table S1. Primers and probes used for rt-PCR assays.**a

| **Target Gene** | **Oligo Designation** | **5' to 3' Nucleotide Sequence** | **Amplicon Size** |
| --- | --- | --- | --- |
| Nm-*ctrA* | F753 | TGT GTT CCG CTA TAC GCC ATT |  |
|  | R846 | GCC ATA TTC ACA CGA TAT ACC | 114 bp |
|  | Pb820ib | (FAM)-AAC CTT GAG CAA "T"CC ATT TAT CCT GAC GTT CT-(SpC6) |  |
| Nm-*sodC* | F351 | GCA CAC TTA GGT GAT TTA CCT GCA T |  |
|  | R478 | CCA CCC GTG TGG ATC ATA ATA GA | 127 bp |
|  | Pb387 | (FAM)-CAT GAT GGC ACA GCA ACA AAT CCT GTT T-(BHQ1) |  |
| Hi-*hpd* #1 | hpdF729 | AGA TTG GAA AGA AAC ACA AGA AAA AGA |  |
|  | hpdR819 | CAC CAT CGGCAT ATT TAA CCA CT | 113 bp |
|  | hpdPbr762ibb | (HEX)-AAA CAT CCA ATC G"T"A ATT ATA G-(SpC6) |  |
| Hi-*hpd* #3 | hpdF822 | GGT TAA ATA TGC CGA TGG TGT TG |  |
|  | hpdR952 | TGC ATC TTT ACG CAC GGT GTA | 151 bp |
|  | Pb896ib | (FAM)-TTG TGT ACA CTC CGT "T"GG TAA AAG AAC TTG CAC-(SpC6) |  |
| Sp-*lytA* | F373 | ACG CAA TCT AGC AGA TGA AGC A |  |
|  | R424 | TCG TGC GTT TTA ATT CCA GCT | 51 bp |
|  | Pb400 | (FAM)-TGC CGA AAA CGC TTG ATA CAG GGA G-(BHQ1) |  |
| NmA-*csaB* | F2531 | AAA ATT CAA TGG GTA TAT CAC GAA GA |  |
|  | R2624 | ATA TGG TGC AAG CTG GTT TCA ATA G | 92 bp |
|  | Pb2591ib | (FAM)-CTA AAA G"T"A GGA AGG GCA CTT TGT GGC ATA AT-(SpC6) |  |
| NmB-*csb* | F737 | GCT ACC CCA TTT CAG ATG ATT TGT |  |
|  | R882 | ACC AGC CGA GGG TTT ATT TCT AC | 169 bp |
|  | Pb839ib | (FAM)-AAG AGA TGG GYA ACA AC "T" ATG TAA TGT CTT TAT TT-(SpC6) |  |
| NmC-*csc* | F478 | CCC TGA GTA TGC GAA AAA AAT T |  |
|  | R551 | TGC TAA TCC CGC CTG AAT G | 77 bp |
|  | Pb495ib | (FAM)-TTT CAA TGC "T"AA TGA ATA CCA CCG TTT TTT TGC-(SpC6) |  |
| NmW-*csw* | F857 | TAT TTA TGG AAG GCA TGG TGT ATG |  |
|  | R964 | TTG CCA TTC CAG AAA TAT CAC C | 129 bp |
|  | Pb907ib | (FAM)-AAA TAT GGA GCG AA"T" GAT TAC AGT AAC TAT AAT GAA-(SpC6) |  |
| NmX*-csxB* | F173 | TGT CCC CAA CCG TTT ATT GG |  |
|  | R237 | TGC TGC TAT CAT AGC CGC C | 66 bp |
|  | Pb196 | (FAM)-TGT TTG CCC ACA TGA ATG GCG G-(BHQ1) |  |
| NmY-*csy* | F787 | TCC GAG CAG GAA ATT TAT GAG AAT AC |  |
|  | R929 | TTG CTA AAA TCA TTC GCT CCA TAT | 146 bp |
|  | Pb1099ib | (FAM)-TAT GGT G"T"A CGA TAT CCC TAT CCT TGC CTA TAA T-(SpC6) |  |

a All primer and probes were sysnthesized at the CDC Biotechnology Core Facility

b Black Hole Quencher (BHQ) at internal “T”
