# Supplementary material for: Development of Real-Time PCR Methods for the Detection of Bacterial Meningitis Pathogens without DNA Extraction
Source: PLoS One. 2016 Feb 1;11(2):e0147765. doi: 10.1371/journal.pone.0147765 (PMC4735509; doi:10.1371/journal.pone.0147765)
Supplement: S2 Table — (DOCX) [file pone.0147765.s002.docx]

**Table S2. Reaction assembly for traditional and two direct real-time PCR methods.**a

| Methods | Reagent | Volume per reaction (µl) |
| --- | --- | --- |
| Traditional (TaqMan) | Taq mix | 12.5 |
|  | PCR Grade H_2_0 | 4.5 |
|  | Forward Primer | 2 |
|  | Reverse Primer | 2 |
|  | Probe | 2 |
|  | Extracted DNA | 2 |
|  | Total Volume | 25 |
| Direct (5x Omni) | 5x OmniTaq mix | 5 |
|  | PEC-2 | 12.5 |
|  | PCR Grade H_2_0 | 2.5 |
|  | Forward Primer | 1 |
|  | Reverse Primer | 1 |
|  | Probe | 1 |
|  | CSF | 2 |
|  | Total Volume | 25 |
| Direct (PerfeCTa) | ToughMix | 12.5 |
|  | PCR Grade H_2_0 | 7.5 |
|  | Forward Primer | 1 |
|  | Reverse Primer | 1 |
|  | Probe | 1 |
|  | CSF | 2 |
|  | Total Volume Volume | 25 |

a A 25-ul PCR reaction was used for the traditional real-time PCR and direct real-time PCR methods.
