# Supplementary material for: Development of Real-Time PCR Methods for the Detection of Bacterial Meningitis Pathogens without DNA Extraction
Source: PLoS One. 2016 Feb 1;11(2):e0147765. doi: 10.1371/journal.pone.0147765 (PMC4735509; doi:10.1371/journal.pone.0147765)
Supplement: S3 Table — (DOCX) [file pone.0147765.s003.docx]

**Table S3. Cycling conditions for the traditional and two direct real-time PCR methods.**

| Methods | Temperature | Time | Cycles |
| --- | --- | --- | --- |
| Traditional (TaqMan) | 50°C | 2 min | 1 |
|  | 95°C | 10 min | 1 |
|  | 95°C | 15 sec | 50a |
|  | 60°C | 1 min |  |
|  |  |  |  |
| Direct (5x Omni and PerfeCTa) | 95°C | 10 min | 1 |
|  | 95°C | 15 sec | 50 a |
|  | 60°C | 1 min |  |
|  |  |  |  |
| Direct (5x Omni)b | 94°C | 5 min | 1 |
|  | 94°C | 30 sec | 50 a |
|  | 55°C | 30 sec |  |
|  | 68°C | 30 sec |  |
|  | 68°C | 1 min | 1 |

**a** Annealing and extension were run for a total of 50 cycles.

b Direct real-time PCR cycling condition for *lytA* and *hpd* #1 tests using 5x Omni.
