# Supplementary material for: Development of Real-Time PCR Methods for the Detection of Bacterial Meningitis Pathogens without DNA Extraction
Source: PLoS One. 2016 Feb 1;11(2):e0147765. doi: 10.1371/journal.pone.0147765 (PMC4735509; doi:10.1371/journal.pone.0147765)
Supplement: S4 Table — (DOCX) [file pone.0147765.s004.docx]

**Table S4. Panel of 25 species representing 14 different genera of bacterial isolates used for cross-reactivity testing.**a

| Species | No. of isolates tested |
| --- | --- |
| *Actinobacillus pleuropneumoniae* | 1 |
| *Aggregatibacter aphrophilus* | 3 |
| *Bordetella pertussis* | 1 |
| *Branhamella catarrhalis* | 1 |
| *Corynebacterium diphtheriae* | 1 |
| *Cryptococcus neoformans* | 1 |
| *Escherichia coli* 016:K1 (L) | 1 |
| *Escherichia coli* 07:K1(L) | 1 |
| *Haemophilus haemolyticus* | 1 |
| *Haemophilus aegyptius* | 2 |
| *Haemophilus influenzae* serotypes a, b, c, d, e, f, NT | 1 of each serotype |
| *Haemophilus parahaemolyticus* | 1 |
| *Haemophilus parainfluenzae* | 6 |
| *Haemophilus species* | 1 |
| *Listeria monocytogenes* | 1 |
| *Neisseria cinerea* | 2 |
| *Neisseria gonorrhoeae* | 1 |
| *Neisseria lactamica* | 11 |
| *Neisseria meningitidis* serogroups E, A, B, B(2+), C, W, X, Y, NG | 1 of each serogroup |
| *Neisseria meningitidis* Z | 2 |
| *Neisseria sicca* | 1 |
| *Neisseria subflava* | 1 |
| *Pseudomonas aeruginosa* | 1 |
| *Salmonella choleraesuis* | 1 |
| *Staphylococcus aureus* | 1 |
| *Streptococcus agalactiae* | 1 |
| *Streptococcus pneumoniae* | 1 |
| Total | 66 |

a Isolates were selected based on their available at the laboratory. Real-time PCR was performed on crude DNA preps for each isolate.
